# Patterns of caesarean section in HIV infected and non-infected women in Malawi: is caesarean section used for PMTCT?

**DOI:** 10.1186/s12884-018-1722-4

**Published:** 2018-04-12

**Authors:** Lyson Tenthani, Joep J. van Oosterhout, Andreas D. Haas, Malango Msukwa, Nozgechi Phiri, Frank Chimbwandira, Kali Tal, Karoline Aebi-Popp, Janne Estill, Olivia Keiser

**Affiliations:** 1 Department of HIV and AIDS, Ministry of Health, Lilongwe, Malawi; 2 Institute of Social and Preventive Medicine, University of Bern, Bern, Switzerland; 3 International Training and Education Center for Health, P.O Box 30369, Lilongwe 3, Malawi; 4 Dignitas International, Zomba, Malawi; 5 Department of Medicine, College of Medicine, Blantyre, Malawi; 6 The Baobab Health Trust, Lilongwe, Malawi; 7 Department of Infectious Diseases, University Hospital Bern, Bern, Switzerland; 8 Institute of Global Health, University of Geneva, Geneva, Switzerland; 9 Institute of Mathematical Statistics and Actuarial Science, University of Bern, Bern, Switzerland

## Abstract

**Background:**

Caesarean section (CS) is not recommended for PMTCT in Malawi HIV Guidelines, contrary to most high-income countries where CS is indicated if viral suppression is sub-optimal pre-delivery. We describe patterns of CS in HIV-infected and uninfected women in Malawi and explored if insight into the use of Elective CS (ECS) for PMTCT could be obtained.

**Methods:**

We used routinely collected data from individual medical records from 17 large health facilities in the central and southern regions of Malawi, from January 2010 to December 2013. We included data from maternity registers from all HIV-positive women, and randomly selected around every fourth woman with negative or unknown HIV status. We used multivariable logistic regressions and cluster-based robust standard errors to examine independent associations of patient- and facility characteristics with CS and ECS.

**Results:**

We included 62,033 women in the analysis. The weighted percentage of women who had a spontaneous vaginal delivery was 80.0% (CI 95% 79.5–80.4%); 2.4% (95% CI 2.3–2.6%) had a vacuum extraction; 2.3% (95% CI 2.2–2.5%) had a vaginal breech delivery; 14.0% (95% CI 13.6–14.4%) had a CS while for 1.3% (95% CI 1.2–1.4%) the mode of delivery was not recorded. Prevalence of CS without recorded medical or obstetric indication (ECS) was 5.1%, (*n* = 3152). Presence of maternal and infant complications and older age were independently associated with CS delivery. HIV-positive women were less likely to have ECS than HIV negative women (aOR 0.65; 95%-CI 0.57–0.74). Among HIV-positive women, those on antiretrovirals (ARV’s) for ≥4 weeks prior to delivery were less likely to have ECS than HIV-positive women who had not received ARVs during pregnancy (aOR 0.81; 95% CI 0.68–0.96).

**Conclusions:**

The pattern of CS’s in Malawi is largely determined by maternal and infant complications. Positive HIV status was negatively associated with CS delivery, possibly because health care workers were concerned about the risk of occupational HIV transmission and the known increased risk of post-operative complications. Our results leave open the possibility that CS is practiced to prevent MTCT given that ECS was more common among women at high risk of MTCT due to no or short exposure to ARV’s.

**Electronic supplementary material:**

The online version of this article (10.1186/s12884-018-1722-4) contains supplementary material, which is available to authorized users.

## Background

In 2014, an estimated 1.1 million people were living with HIV in Malawi, including 130,000 children under fifteen [1]. Mother-to-child transmission (MTCT) is the most common cause of infection in children. Without intervention, risk of MTCT is estimated to be 5–10% over the course of pregnancy, 10–20% during labour, and 10–20% during breastfeeding; overall 30–45% of infants born to mothers with HIV will contract the virus [2]. With effective antiretroviral therapy (ART), the risk of MTCT can be reduced to less than 5% [3].

In Malawi the total fertility rate is estimated to be between 4.4 and 5.7 children per woman. High ratios of maternal mortality (460–680 per 100,000 live births) and neonatal mortality (27 per 1000 live births) have been recorded. The contraceptive prevalence rate varies between 44.4% (sexually active unmarried women) and 59.2% (married women) [4]. Maternal mortality is believed to be strongly impacted on by the HIV epidemic, with a steep increase between 1992 and 2000 and a sharp decline since the start of free ART provision in 2004 [4].

In this context, Malawi implemented a test and treat policy (“Option B+”) to facilitate access to ART for all HIV-infected pregnant and breastfeeding women [4]. The policy was adopted by other low-income countries and is now recommended by the World Health Organization [5]. Under Option B+, women are diagnosed with HIV during routine testing in antenatal care and they initiate ART within one week of diagnosis [4]. In 2011, Option B+ was implemented as Malawi’s National PMTCT policy and the ART coverage among pregnant women increased strongly [6]. However, even with Option B+, a substantial proportion of women are likely not to be fully virologically suppressed when around 20% of pregnant women are not tested for HIV [7]; almost 20% discontinue treatment within the first months [8, 9]; and about 30% adhere to ART inadequately during pregnancy, with young age and Option B+ indication for ART as risk factors [10]. Overall, 9% of women who are on ART during pregnancy do not achieve adequate virological suppression at delivery [11].

Caesarean section (CS) can be used to reduce the risk of intrapartum HIV transmission [12–14]. In high-income countries CS is recommended for women with > 400 HIV RNA copies/ml at the time of delivery [15, 16]. In resource constrained settings like Malawi, elective caesarean section (ECS; defined here as a CS without documented obstetric or medical indication) is not a recommended PMTCT strategy [17] as the increased risk of morbidity and mortality that is associated with ECS is expected to outweigh its HIV prevention benefits [18, 19]. In addition, resources and capacity to perform CS are limited in low-income countries like Malawi [19, 20]. Despite this, it is possible that health care workers and pregnant women in Malawi opt for an ECS in circumstances where MTCT risk is known or believed to be high.

We describe the prevalence and pattern of indications of CS among deliveries of HIV positive and HIV negative women in Malawi. We also explore our data for the potential practice of using ECS for PMTCT.

## Methods

### Data sources

We entered routinely collected data from individual medical records in 17 large health facilities in the central and southern regions of Malawi, from January 2010 to December 2013. The selected facilities were among 20 study facilities that participate in the Umoyo+ study (http://aidsinfo.unaids.org/). Two of the 20 facilities were excluded because no CS’s were done, another one because of missing data. In the Malawi government health care system, observations from labour and delivery are recorded on labour charts and summarized in a maternity register. These standard monitoring and evaluation tools capture demographic characteristics, obstetrical history, and observations on the delivery and infant. Theatre registers document detailed information about CS (i.e. indication, duration, observations and outcome of the procedure). At each health facility, we included data from maternity registers from all HIV positive women and from a randomly selected approximately one quarter of women with negative or unknown HIV status. We describe the sampling strategy in more detail in the Additional file 1: Appendix 1. In case the indication for CS could not be determined from the maternity register, we extracted relevant information from labour charts and theatre registers.

### Definitions

Health care workers classified the mode of delivery either as CS or as vaginal delivery (including assisted vaginal deliveries that use vacuum and forceps extraction). The main outcomes of the study were CS and ECS. The delivery was defined as CS when it was conducted through CS irrespective of the reason for the procedure. A CS was classified as ECS if it was conducted electively without documentation of an obstetric or medical indication. In women with a previous CS, the current CS was counted as ECS only if there was no trial of vaginal delivery for the current delivery with no previous history of CS (if a previous CS was the indication for the current CS, this will be reported according to local guidelines). HIV status was positive if a woman was admitted for labour and delivery with documentation of a positive HIV test result or if she was on ART, or if she had a positive test result obtained between the onset of labour and the period immediately after delivery. A woman was classified as HIV negative if she had a negative test result in that same period or if she had a documented negative test result during the current pregnancy. HIV unknown status was assigned if testing was not documented, if it was documented as not done, or if test results were inconclusive or missing.

### Statistical analysis

**Fig. 1:**
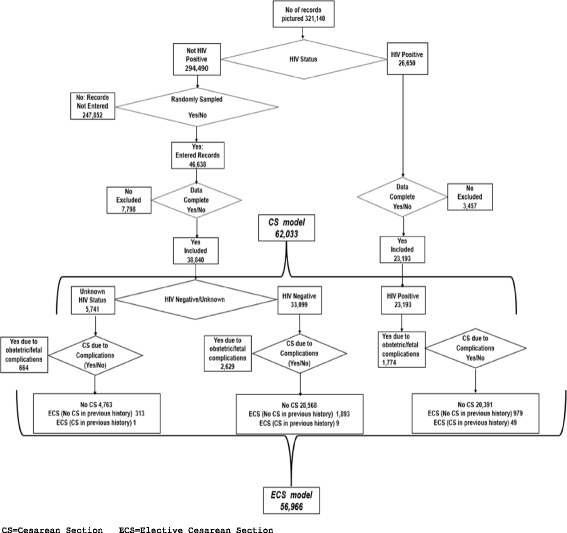
Flow chart showing the patients’ records included in the study and the specific model according to their HIV status and mode of delivery

We provide patterns of indications for CS among HIV positive, unknown and negative women using descriptive statistics. To examine independent associations between CS or ECS and patient and facility characteristics, we used multivariable logistic regression analyses, with cluster-based robust standard errors adjusting for clustering of patients within facilities. We considered the following explanatory variables: year of admission, maternal complications (hemorrhage, obstructed/prolonged labour, pre-eclampsia, maternal sepsis, ruptured uterus, others when unspecified, unknown if nothing was recorded), infant complications (prematurity, low birth weight, asphyxia, other if unspecified, unknown if nothing was recorded), HIV status (negative, positive, unknown), and ARV use during pregnancy (no ARVs, ARVs for < 4 weeks, ARVs for≥4 weeks), number of deliveries (0, 1, 2–3, > 3), singleton (yes/no), age category (< 20, 20–24, 25–29, 30–34, > 34 years), facility ownership (government or Christian Health Association (CHAM)) and location (urban or rural). We ran 4 models each to examine predictors of CS and predictors of ECS. We included all women in the first CS model; women with unknown HIV status in the second, HIV negative women in the third model and HIV positive women in the fourth. In the four ECS models, we excluded women who had a previous CS and failed trial of vaginal delivery and women with a recorded maternal or infant complication (as these women were not at risk of ECS) (Fig. 1). In all analyses HIV negative women were weighted by the inverse of the probability that the observation was included because of the sampling design. HIV positive women were assigned a weight of 1 as data from all HIV positive women was analyzed (see sampling strategy description in Additional file 1: Appendix 1). We therefore report weighted prevalence rates throughout. All data were analyzed with STATA software (Version 13.1, Stata Corporation, Texas USA.

## Results

### Characteristics of study participants

Out of 62,033 women included in the analysis 56,292 (90.7%) had a known HIV status and 5741 (9.3%) had an unknown HIV status. Among women with known HIV status 23,193 were HIV positive and 33,099 were HIV negative, resulting in a weighted HIV prevalence of 9.7%. (95% CI 9.3–10.1%). Characteristics of study participants stratified by HIV status are shown in Table 1. Their median age was 25 years (interquartile range [IQR] 20–30). Women had a median of one delivery (IQR 1–3). The majority of women (89.6%, 55,583) received care at government owned facilities and 10.4% (6450) were managed in faith-based clinics. About 76% of the women (47,215) attended clinics in rural settings and 24% (14,818) were seen at urban clinics.

**Table 1:** Characteristics of study participants by HIV status

Item		All women (*n* = 62,033)		Women with unknown HIV status (*n* = 5741)		HIV negative women (*n* = 33,099)		HIV positive women (*n* = 23,193)	
		Total	Weighted %	Total	Weighted %	Total	Weighted %	Total	Weighted %
Year of admission	2010	10,526	20.8	675	18.4	4272	20.9	5579	24.1
	2011	16,216	30.9	1647	38.2	7056	29.4	7513	32.4
	`2012	16,749	22.6	1888	23.6	9889	22.5	4972	21.4
	2013	18,542	25.8	1531	19.9	11,882	27.2	5129	22.1
Maternal complication	None	52,992	86.8	4674	82.4	28,932	88.0	19,386	83.6
	Hemorrhage	1566	2.0	173	2.9	621	1.7	772	3.3
	OPL	2899	4.3	312	5.0	1413	4.7	1175	5.1
	Preeclampsia	511	0.8	78	1.2	260	0.7	173	0.7
	Sepsis	101	0.1	13	0.3	21	0.1	67	0.3
	RU	161	0.2	28	0.5	68	0.2	65	0.3
	Unspecified	3275	5.0	386	6.4	1610	4.7	1278	5.5
	Unknown	528	0.7	77	1.4	174	0.5	277	1.2
Infant complication	None	51,017	85.8	4691	82.1	28,892	87.6	17,435	75.2
	Prematurity	2609	3.6	281	4.7	1163	3.3	1156	5.0
	LBW	2166	3.0	261	4.8	844	2.5	1073	4.6
	Asphyxia	2140	3.4	246	3.9	1158	3.3	732	3.2
	Unspecified	1683	2.6	197	3.3	811	2.4	675	2.9
	Unknown	2418	1.6	65	1.2	231	0.8	2122	9.1
No of previous deliveries	0	14,446	29.1	1738	30.6	10,125	30.8	2583	11.1
	1	12,554	21.2	1138	19.9	7314	21.8	4102	17.7
	2–3	19,780	28.0	1505	26.2	8856	26.9	9419	40.6
	> 3	14,554	20.5	1180	20.4	6523	19.6	6851	29.5
	Unknown	699	1.2	180	2.9	281	0.9	238	1.0
Age in years	< 20	11,191	23.0	1428	26.3	8217	24.4	1546	6.7
	20–24	17,485	31.0	1738	30.5	10,491	32.0	5256	22.7
	25–29	14,003	20.0	1035	17.1	6374	19.5	6595	28.4
	30–34	10,960	13.9	740	12.5	4389	12.9	58,31	25.1
	> 34	7288	10.2	583	9.8	3094	9.6	3610	15.6
	Unknown	1106	1.9	217	4.0	534	1.6	355	1.5
Singleton	Yes	59,862	96.6	5519	96.1	32,068	96.7	22,274	96.0
	No	2171	3.4	222	3.9	1031	3.3	919	4.0
ARVs during pregnancy	None, HIV+	11,938	4.3	N/A				11,938	51.5
	<4wks	2716	1.0					2716	11.7
	≥4wks	8539	3.1					8539	36.8
	None, HIV-	38,840	91.6					N/A	
Facility ownership	Government	55,583	89.6	5476	95.3	29,447	88.1	20,660	89.1
	CHAM	6450	10.4	265	4.7	3652	11.9	2533	10.9
Facility location	Rural	47,215	80.4	4936	86.2	26,660	80.8	15,619	67.3
	Urban	14,818	19.6	805	13.8	6439	19.2	7574	32.7

*aOR* adjusted odds ratio, *OPL* obstructed/prolonged labor, *RU* Ruptured Uterus, *LBW* Low Birth Weight, *ARVs* antiretrovirals, *CHAM* Christian Health Association of Malawi

### Maternal and infant complications

The proportion of women with maternal complications was 12.5%(95% CI 12.1–12.9%). Maternal complications occurred more frequently among HIV positive (15.2%; 95% CI 14.8–15.7%) than among HIV negative women (11.5%; 95% CI 11.1–11.9%). Among women with unknown HIV status the corresponding percentage was 16.2% (95% CI 15.2–17.3%). The percentage of infants who had complications was 14.5% (95% CI 14.1–14.8%). Infant complications were more common among HIV exposed infants (25.1%, CI 24.5–25.7%) than among infants born to mothers with negative (12.7%, CI 12.3–13.1%) or unknown HIV status (18.3%, CI 17.2–19.5%). The distribution of specific maternal and infant complications by HIV status is shown in Table 1.

### Mode of delivery

The percentage of women who had a spontaneous vaginal delivery was 80.0% (CI 95% 79.5–80.4%); 2.4% (95% CI 2.3–2.6%) had vacuum extraction; 2.3% (95% CI 2.2–2.5%) had a vaginal breech delivery; 14.0% (95% CI 13.6–14.4%) had CS while for 1.3% (95% CI 1.2–1.4%) of the women mode of delivery was not recorded.

Overall, prevalence of CS among women with unknown HIV status was significantly higher than among deliveries of HIV negative women, which in turn was significantly higher than among deliveries in HIV positive women (HIV unknown 16.2%, 95% CI 15.1–17.3%; HIV negative 13.8%, 95% CI 13.3–14.2%; HIV positive 12.1%, 95% CI 11.7–12.5%). The distribution of CS according to individual and facility-level characteristics stratified by HIV status is shown in Table 2. Among women with a recorded complication 64.4% (95% CI 63.9–64.9%) had a CS delivery compared to 6.8% (95% CI 6.7–6.9%) in women without a recorded complication. The prevalence of elective CS without recorded medical or obstetric indication (ECS) was 5.1% (95% CI 4.9–5.3%).

**Table 2:** Proportion of women who delivered through C-section according to baseline characteristics

Item		All Women (n = 62,033)	Women with unknown HIV status (n = 5741)	HIV negative women n = 33,099)	HIV positive women n = 23,193
Year of admission	2010	15.2 (14.2–16.3)	17.2 (14.3–20.7)	15.1 (13.9–16.4)	13.7 (12.8–14.6)
	2011	13.7 (12.9–14.5)	13.4 (11.7–15.4)	13.8 (12.9–14.9)	13.3 (12.5–14.1)
	2012	13.4 (12.8–13.9)	18.2 (16.6–20.1)	12.8 (12.2–13.5)	9.6 (8.8–10.5)
	2013	13.8 (13.3–14.4)	18.1 (16.2–20.1)	13.5 (12.9–14.1)	11.0 (10.2–11.9)
Maternal complication	None	6.6 (6.3–6.9)	6.3 (6.1–6.6)	6.8 (6.6–6.9)	5.1 (4.8–5.4)
	Hemorrhage	29.5 (26.5–32.8)	34.7 (32.0–37.6)	30.2 (28.7–31.7)	19.0 (16.4–22.0)
	OPL	68.2 (65.8–70.4)	68.0 (65.9–70.0)	69.2 (68.2–70.1)	60.9 (58.0–63.6)
	Preeclampsia	60.2 (55.0–65.2)	64.8 (60.4–69.1)	61.2 (58.7–63.6)	39.3 (32.3–46.8)
	Sepsis	16.5 (7.3–33.3)	5.1 (2.1–11.5)	30.7 (23.4–39.2)	6.0 (2.2–14.9)
	RU	87.4 (78.5–93.0)	84.7 (78.6–89.4)	90.7 (87.6–93.1)	72.3 (60.2–81.9)
	Unspecified	75.7 (73.5–77.8)	77.0 (75.3–78.6)	77.2 (76.3–78.0)	61.4 (58.7–64.1)
	Unknown	33.0 (27.6–38.8)	31.9 (28.1–36.1)	36.6 (33.8–39.4)	20.6 (16.2–25.8)
Infant complication	None	12.4 (12.0–12.8)	13.9 (13.5–14.3)	12.3 (12.1–12.4)	11.2 (10.7–11.7)
	Prematurity	17.0 (15.1–19.1)	21.7 (19.9–23.7)	16.1 (15.3–17.0)	15.2 (13.3–17.4)
	LBW	20.8 (18.4–23.4)	16.1 (14.5–17.8)	23.0 (21.9–24.1)	18.0 (15.8–20.4)
	Asphyxia	29.2 (26.7–31.8)	40.0 (37.5–42.5)	26.8 (25.8–27.8)	30.2 (27.0–33.6)
	Unspecified	32.4 (29.5–35.5)	36.8 (34.2–39.5)	31.8 (30.6–33.1)	29.2 (25.9–32.7)
	Unknown	15.7 (12.9–19.1)	15.5 (12.5–19.0)	31.2 (29.1–33.4)	2.7 (2.1–3.5)
No of previous deliveries	0	16.3 (15.5–17.1)	16.2 (15.5–16.9)	16.2 (16.0–16.5)	18.7 (17.3–20.3)
	1	14.2 (13.3–15.0)	15.9 (15.1–16.8)	14.0 (13.7–14.3)	12.6 (11.6–13.6)
	2–3	13.3 (12.6–14.0)	17.8 (17.0–18.5)	12.7 (12.5–13.0)	11.6 (11.0–12.3)
	> 3	11.2 (10.5–12.1)	13.8 (13.0–14.6)	11.0 (10.7–11.3)	9.9 (9.2–10.6)
	Unknown	17.4 (14.1–21.3)	20.2 (18.0–22.7)	16.2 (14.6–17.9)	14.3 (10.4–19.3)
Age in years	< 20	14.3 (13.5–15.1)	13.3 (12.6–14.0)	14.5 (14.2–14.8)	13.6 (12.0–15.5)
	20–24	14.6 (13.9–15.4)	18.5 (17.8–19.3)	14.2 (13.9–14.4)	12.3 (11.4–13.2)
	25–29	14.6 (13.8–15.5)	18.7 (17.8–19.6)	14.4 (14.1–14.7)	11.8 (11.1–12.6)
	30–34	12.1 (11.2–13.0)	13.7 (12.8–14.7)	11.8 (11.4–12.2)	11.9 (11.1–12.8)
	> 34	12.6 (11.5–13.8)	15.3 (14.2–16.5)	12.2 (11.8–12.7)	11.9 (10.9–13.0)
	Unknown	13.6 (11.1–16.4)	15.3 (13.6–17.3)	13.0 (11.9–14.1)	12.4 (9.3–16.3)
Singleton	Yes	13.7 (13.4–14.1)	16.0 (15.7–16.4)	13.6 (13.4–13.7)	11.7 (11.3–12.2)
	No	20.4 (17.9–23.0)	19.7 (17.8–21.8)	20.4 (19.5–21.4)	20.8 (18.2–23.4)
ARVs during pregnancy	None	11.7 (11.1–12.3)	N/A		11.7 (11.1–12.3)
	<4wks	12.4 (11.3–13.7)			12.4 (11.3–13.7)
	≥4wks	12.5 (11.9–13.2)			12.5 (11.9–13.3)
	HIV- & Unknown	14.1 (13.7–14.6)			N/A
Facility ownership	Government	13.8 (13.4–14.2)	16.2 (15.8–16.5)	13.5 (13.4–13.7)	12.2 (11.8–12.7)
	CHAM	15.3 (14.1–16.7)	16.6 (15.0–18.4)	15.7 (15.2–16.1)	11.3 (10.1–12.5)
Facility location	Rural	13.6 (13.4–13.7)	16.0 (15.6–16.4)	13.3 (13.1–13.4)	11.4 (10.9–11.9)
	Urban	15.7 (15.4–16.0)	17.3 (16.3–18.3)	15.9 (15.5–16.2)	13.6 (12.9–14.4)
Totals		14.0 (13.6–14.4)	16.2 (15.1–17.3)	13.8 (13.3–14.2)	12.1 (9.1–15.1)

*OPL* obstructed/prolonged labor, *RU* Ruptured Uterus, *CHAM* Christian Health Association of Malawi, *LBW* Low Birth Weight, *aOR* adjusted odds ratio, *ARVs* antiretrovirals

### Associations of individual and facility level characteristics with CS

HIV positive women were less likely to have CS compared to HIV negative women (aOR 0.60; 95%-CI 0.51–0.71) while those with unknown HIV status had similar odds of CS as HIV negative women (aOR 1.01; 96% CI 0.84–1.20). Table 3 shows the results from the multivariable logistic regression analyses of variables associated with CS. Presence of maternal complications, infant complications and older age were independently associated with CS delivery in each of the three groups. Twin delivery and delivering for the first time were associated with a higher probability of CS in all groups. Year of admission was independently associated with a lower odds of CS after 2010 only in HIV negative women.

**Table 3:** Individual and facility-level factors associated with C-Section among women delivering in 17 large health facilities in Malawi

Item		aOR (95% CI) All Women (55,443)	*p*	aOR (95% CI) Women with Unknown HIV status (*n* = 5302)	*P*	aOR (95% CI) HIV- Women (n = 31,978)	*p*	aOR (95% CI) HIV+ Women (*n* = 20,436)	*P*
Year of admission	2010	1	0.07	1	0.09	1	0.02	1	0.06
	2011	0.71 (0.50–1.01)		0.58 (0.37–0.91)		0.71 (0.50–1.00)		1.00 (0.73–1.38)	
	2012	0.65 (0.45–0.93)		0.71 (0.42–1.19)		0.61 (0.43–0.88)		0.83 (0.56–1.22)	
	2013	0.67 (0.49–0.92)		0.67 (0.43–1.07)		0.65 (0.48–0.87)		1.04 (0.73–1.49)	
Maternal Obstetric Complication	None	1	< 0.01	1	< 0.01	1	< 0.01	1	< 0.01
	Hemorrhage	5.85 (3.12–10.97)		7.02 (4.26–11.58)		5.80 (3.03–11.12)		4.25 (1.72–10.47)	
	OPL	32.23 (17.15–60.57)		29.43 (17.75–48.80)		33.61 (16.99–66.47)		29.88 (11.89–75.13)	
	Preeclampsia	21.76 (10.32–45.86)		27.18 (14.18–52.09)		22.14 (9.83–49.88)		11.23 (4.56–27.68)	
	Sepsis	4.25 (1.30–13.85)		1.35 (0.14–13.18)		8.13 (1.57–42.16)		1.20 (0.36–4.02)	
	RU	160.25 (75.41–340.51)		141.95 (34.98–576.08)		186.48 (66.45–523.27)		94.91 (43.33–207.89)	
	Others	52.85 (31.25–89.39)		51.75 (35.26–75.97)		55.-81 (33.24–93.69)		38.01 (15.13–95.52)	
Infant Complication	None	1	0.01	1	< 0.01	1	< 0.01	1	< 0.01
	Prematurity	0.99 (0.77–1.29)		1.12 (0.61–2.05)		0.95 (0.61–1.46)		1.11 (0.87–1.42)	
	LBW	1.40 (1.07–1.83)		0.77 (0.38–1.58)		1.75 (1.28–2.39)		1.09 (0.77–1.53)	
	Asphyxia	1.86 (1.29–2.67)		3.02 (1.83–4.96)		1.65 (1.08–2.53)		1.92 (1.33–2.76)	
	Others	1.59 (1.15–2.19)		2.20 (1.07–4.52)		1.53 (1.10–2.11)		1.14 (0.73–1.78)	
No of previous deliveries	0	1	< 0.01	1	0.31	1	< 0.01	1	< 0.01
	1	0.83 (0.74–0.93)		0.84 (0.63–1.12)		0.84 (0.74–0.95)		0.61 (0.49–0.76)	
	2–3	0.68 (0.56–0.83)		0.79 (0.55–1.13)		0.68 (0.55–0.84)		0.50 (0.40–0.62)	
	> 3	0.52 (0.41–0.66)		0.72 (0.50–1.05)		0.51 (0.39–0.67)		0.35 (0.26–0.45)	
Age (years)	< 20	1	< 0.01	1	0.02	1	0.01	1	< 0.01
	20–24	1.28 (1.12–1.47)		1.75 (1.25–2.44)		1.20 (1.04–1.39)		1.37 (1.12–1.67)	
	25–29	1.56 (1.27–1.93)		1.96 (1.23–3.11)		1.49 (1.16–1.92)		1.72 (1.34–2.21)	
	30–34	1.44 (1.15–1.81)		1.66 (0.99–2.78)		1.35 (1.03–1.78)		1.98 (1.50–2.61)	
	> 34	1.63 (1.34–1.99)		1.62 (1.00–2.64)		1.57 (1.21–2.03)		2.24 (1.75–2.85)	
Singletons	Yes	1	< 0.01	1	0.35	1	0.03	1	0.04
	No	1.49 (1.10–2.03)		1.31 (0.74–2.33)		1.51 (1.05–2.16)		1.58 (1.03–2.43)	
ARVs during Pregnancy	None	N/A		N/A		N/A		1	0.83
	<4wks	N/A		N/A		N/A		0.98 (0.72–1.32)	
	≥4wks	N/A		N/A		N/A		0.96 (0.81–1.12)	
Facility Ownership	Govt.	1	0.90	1	0.57	1	0.36	1	0.77
	CHAM	1.42 (0.65–3.13)		1.27 (0.55–2.94)		1.48 (0.64–3.41)		1.09 (0.62–1.90)	
Facility Location	Rural	1	0.27	1	0.26	1	0.13	1	0.23
	Urban	1.39 (0.91–2.12)		1.19 (0.88–1.62)		1.47 (0.89–2.41)		1.16 (0.91–1.46)	

*OPL* obstructed/prolonged labor, *RU* Ruptured Uterus, *CHAM* Christian Health Association of Malawi, *LBW* Low Birth Weight, *aOR* adjusted odds ratio, *ARVs* antiretrovirals

### Associations of individual and facility level characteristics with ECS

Results from the multivariable logistic regression analyses of variables associated with ECS delivery among all women, HIV positive women, those with unknown HIV status and HIV negative women are presented in Table 4. HIV positive women were less likely to have ECS compared to HIV negative women (aOR 0.65; 95%-CI 0.57–0.74). Among HIV positive women, those who were on ARV’s for ≥4 weeks prior to delivery were less likely to have ECS than HIV positive women who had not received ARVs during pregnancy. Older age and delivery in an urban facility were associated with higher odds of ECS in all groups. First deliveries and non-singleton deliveries were independently associated with an increased odds of ECS in all groups except in women with HIV unknown status. Delivery in 2010, was independently associated with ECS, except in HIV positive women.

**Table 4:** Individual and facility-level factors associated with Elective Caesarean Section

Item		aOR (95% CI) All Women (55,443)	*p*	aOR (95% CI) Women with Unknown HIV status (*n* = 4764)	*p*	aOR (95% CI) HIV- Women (*n* = 29,747)	*p*	aOR (95% CI) HIV+ Women (n = 20,932)	*p*
Year of Admission	2010	1	0.03	1	0.01	1	0.02	1	< 0.09
	2011	0.63 (0.42–0.94)		0.47 (0.29–0.76)		0.64 (0.43–0.96)		0.84 (0.55–1.30)	
	2012	0.55 (0.34–0.88)		0.61 (0.31–1.22)		0.53 (0.33–0.86)		0.55 (0.28–1.06)	
	2013	0.53 (0.35–0.83)		0.40 (0.21–0.78)		0.54 (0.36–0.82)		0.68 (0.39–1.19)	
No of deliveries	0	1	< 0.01	1	0.11	1	< 0.01	1	< 0.01
	1	0.76 (0.66–0.87)		0.74 (0.57–0.96)		0.77 (0.66–0.90)		0.59 (0.46–0.75)	
	2–3	0.65 (0.51–0.82)		0.68 (0.42–1.11)		0.65 (0.51–0.83)		0.49 (0.38–0.63)	
	> 3	0.46 (0.34–0.63)		0.68 (0.40–1.16)		0.45 (0.31–0.65)		0.31 (0.22–0.43)	
Age (years)	< 20	1	< 0.01	1	< 0.01	1	< 0.01	1	< 0.01
	20–24	1.38 (1.18–1.61)		2.07 (1.42–3.03)		1.29 (1.09–1.53)		1.46 (1.12–1.91)	
	25–29	1.68 (1.35–2.09)		2.11 (1.21–3.67)		1.60 (1.25–2.06)		1.86 (1.33–2.60)	
	30–34	1.46 (1.10–1.92)		1.44 (0.81–2.54)		1.37 (1.00–1.88)		2.19 (1.40–3.43)	
	> 34	1.68 (1.24–2.27)		1.93 (1.07–3.50)		1.58 (1.08–2.32)		2.26 (1.57–3.27)	
Singletons	Yes	1	< 0.01	1	0.13	1	< 0.01	1	< 0.01
	No	1.98 (1.55–2.54)		1.48 (0.90–2.46)		2.06 (1.54–2.77)		2.27 (1.51–3.41)	
ARVs during Pregnancy	None	N/A		N/A		N/A		1	0.04
	<4wks	N/A		N/A		N/A		0.96 (0.73–1.27)	
	≥4wks	N/A		N/A		N/A		0.81 (0.68–0.96)	
Facility Ownership	Government	1	0.69	1	0.47	1	0.72	1	0.80
	CHAM	1.63 (0.69–3.83		1.19 (0.74–1.91)		1.75 (0.72–4.27)		1.08 (0.59–1.99)	
Facility Location	Rural	1	0.03	1	0.01	1	0.03	1	< 0.01
	Urban	1.95 (1.06–3.56)		1.52 (1.17–1.98)		2.02 (1.05–3.86)		2.15 (1.27–3.64)	

*CHAM* Christian Health Association of Malawi, *aOR* adjusted odds ratio, *ARVs* antiretrovirals

## Discussion

We found that the prevalence of CS in deliveries in 17 large health facilities in Malawi was 14.0% and was higher among deliveries of HIV negative women compared to HIV positive women. The 14% CS prevalence found in our study is almost three times higher than the 5% last reported in the 2010 nationally representative study [21, 22] and the 6.2% reported in a multinational study in sub-Saharan Africa [23]. However this can be explained by our exclusion of deliveries taking place in primary health facilities where CS services are not offered.

CS delivery was strongly associated with presence of maternal or infant complications. As expected, maternal and infant complications were more common among HIV positive women than HIV negative women, while those with unknown HIV status had similar frequency of complications to HIV positive women (possibly due to undiagnosed HIV among those with unknown status). We also found that among HIV positive women the chance of ECS was higher in those who had received less than 4 weeks or no exposure to ARVs during pregnancy. As in other studies the adjusted odds of a CS delivery increased with age and almost doubled among HIV positive women aged above 29 years compared to those below 20 years [24–27].

Our data suggest that patterns of CS in Malawi are also influenced by other factors than maternal and fetal obstetric conditions. The lower frequency of CS among HIV positive women in spite of their higher maternal and infant complication rates could be explained by strict CS indication setting by health care workers in recognition of the increased post-operative morbidity and mortality [28–30]. Clinicians may also be reluctant to perform ECS in HIV infected women due to a perceived risk of occupational HIV transmission [31]. Stigma and discrimination related to HIV infection may also play a role on health care workers’ decision making [32]. On the other hand, we observed that among HIV positive women the prevalence of CS and ECS was higher in those who had no or less than 4 weeks of exposure to ARVs (i.e. were at high MTCT risk) than in women with longer ARV drug usage in pregnancy. This suggests that high MTCT risk is being considered as an indication for CS in Malawian clinical practice.

We are not aware of other publications that assessed why women or health care workers opt for an ECS in sub-Saharan Africa. Most of the recent publications focused on the effect of early ART and Option B+ on PMTCT [33, 34]. A recent systematic review [35] assessed the risks and benefits of ECS in women with HIV. Of 36 studies included, only 3 were done in sub-Saharan Africa. Two of these studies described risk factors for MTCT [36, 37]. Unger et al. analyzed the use of CS over time, and assessed postpartum morbidity and mortality; but they did not discuss why the use of CS increased over time [38].

Our data showed that ECS was more frequent among women who delivered in urban than rural facilities. Several studies observed a higher prevalence of CS in urban settings and this was mostly driven by demands from mothers or by provider preferences [20, 32, 39] although it is also possible that the difference reflects the larger availability of surgery facilities in urban areas. The reason why this difference was only observed in analyses of ECS and not of all CS, may be due to the fact that demands from mothers and provider preferences are not formally accepted indications for CS in guidelines, thus are not reported in registers and delivery charts or due to the fact that knowledge about the potential use of CS for MTCT is higher in urban areas.

Some studies have shown a declining trend in CS upon wider availability of ARVs [40]. We also saw a reduction in CS and ECS deliveries after 2010, but remarkably not in HIV positive women, suggesting that other factors than increased ART coverage determined this [6].

The study has several strengths and limitations. It is based on a large dataset and includes health facilities in different regions in Malawi. Generalization of the results of this study needs to be considered with care because the data were from large health facilities only. Another limitation is that we used routinely collected medical records which may have affected data quality. The proportion of missing data from explanatory variables was below 5%, except for infant complications (Table 1) and we believe it is unlikely that data were systematically missing for particular groups. In our study, the HIV status variable could be determined by HIV test results that became available before the onset of labour and immediately after delivery. In the latter case HIV status could not have impacted on a CS/ECS decision. Because all HIV testing in Malawi is with rapid point-of-care tests that provide a result within 30 min, most results would have been obtained before delivery, but we do not have data to document this. Further, we assumed that CS was elective when no obstetric indication was recorded, while there could simply be missing obstetric data. We limited this as much as possible by complimenting maternity register data with information from labour charts and theatre registers.

We did not have data on ART adherence or treatment interruptions. Health care workers may have considered CS more frequently in patients with poorer adherence because they know that suboptimal adherence impairs the preventive effect of PMTCT.

## Conclusions

We have shown that patterns of CS in Malawi are largely determined by maternal and infant complications. Positive HIV status was inversely associated with CS delivery, possibly related to health care workers’ perceived risk of occupational HIV transmission and the recognition of the known higher rate of post-operative complications. Our results leave open the possibility that CS is practiced as a means of PMTCT in Malawi as we found that ECS was significantly more common among women with a high risk of MTCT due to no or short exposure to ARVs in pregnancy in a setting with limited access to viral load testing. Further studies are needed to confirm our findings, including surveys among providers of CS services and qualitative research involving patients and practitioners about factors related ECS decisions including stigma and wealth.

## Additional file

Additional file 1: Appendix 1. Word document describing the sampling strategy used in the study. (DOCX 24 kb)

## Abbreviations

aOR: adjusted odds ratio
ART: Antiretroviral therapy
ARVs: Antiretrovirals
CHAM: Christian Health Association of Malawi
CI: Confidence interval
CS: Caesarean section
ECS: Elective caesarean section
IQR: Interquartile range
I-TECH: International Training and Education Center for Health
LBW: Low Birth Weight
MOH: Ministry of Health
MTCT: Mother to child transmission
OPL: Obstructed/prolonged labour
PEER: Partnerships for Enhanced Engagement in Research
PMTCT: Prevention of mother to child transmission
