# Supplementary material for: Patterns of caesarean section in HIV infected and non-infected women in Malawi: is caesarean section used for PMTCT?
Source: BMC Pregnancy Childbirth. 2018 Apr 12;18:95. doi: 10.1186/s12884-018-1722-4 (PMC5897999; doi:10.1186/s12884-018-1722-4)
Supplement: Supplementary file 1 — Appendix 1. Word document describing the sampling strategy used in the study. (DOCX 24 kb) [file 12884_2018_1722_MOESM1_ESM.docx]

**Appendix 1**

**Description of the sampling strategy and weighting of records of women who were HIV uninfected or whose HIV status was unknown**

Our dataset was constructed by directly entering all the records from the maternity registers before the implementation of Option B+ (January 2010-December 2011). During the option B+ period (January 2012-December 2014) the dataset was constructed by photographing the maternity registers and entering the data manually into an electronic database. Each page of the paper register, which normally consists of 10 individual records, was photographed as one image. We entered all records of HIV infected women, as well as a random sample of records of women who were HIV uninfected or and those with unknown status*.*

We applied two different methods for sampling the records of HIV negative women and women with unknown HIV status, depending on the time period.

- During the time period before the implementation of the Option B+ PMTCT strategy, we defined the proportion of records with negative or unknown HIV status that were sampled to be equal to the HIV prevalence in each site.
- During the time period starting from the implementation of the Option B+ PMTCT strategy, we aimed to sample 20% of all records with negative or unknown HIV status in each site. For simplicity, we only sampled full images, containing one full page from the paper register. We only considered images not containing any positive records: images with at least one positive record were excluded from the sampling. Therefore, the actual number of images that needed to be sampled depended on the HIV prevalence and distribution of positive record across the pages.

Because of the design of the sampling strategy and incomplete pages, there was some variability in the actual proportion of all non-positive records that were sampled. Of the records sampled before the implementation of Option B+, the proportion of non-positive records sampled was within a margin of 0.1 percentage point from the HIV prevalence, with the exception of two sites where the sampling probability was lower than the HIV prevalence. During the Option B+ period, the final proportion of HIV negative records that were sampled ranged between 15% and 23% across the sites.

The inverse probability weights were calculated for each site and time period (pre Option B+ and Option B+) separately. The weight *w_ij_* for site *i* and time period *_j_* was defined as

*w_ij_* = *N_ij-_*/*S_ij-_* ,

where *S_ij-_* is the number of sampled non-positive records, and *N_ij-_* the total number of non-positive records in the corresponding site and time period.

Table A1 below shows the sampling strategy and the calculated weights.

***Table A1 Details of sampling strategy and weights calculations***

| **Bplus Period** | | | | | | | | | | | | | |
| --- | --- | --- | --- | --- | --- | --- | --- | --- | --- | --- | --- | --- | --- |
| **No of Images** | | | | **Expected Records (10 expected records on each image)** | | | | | | |  |  |  |
| Site | Only non-positive records | At least one positive record | Total no of images | On non-positive Images | On positive images | On total images | positive records on positive images | non-positive records on positive images | Total non-positive (approximated) | Total women expected (=G) | Sampled negative and unknown records | Probability of being selected | Weight |
| 1 | 269 | 384 | 653 | 2,690 | 3,840 | 6,530 | 575 | 3,265 | 5,955 | 6,530 | 1096 | 0.18 | 5.4 |
| 2 | 722 | 191 | 913 | 7,220 | 1,910 | 9,130 | 186 | 1,724 | 8,944 | 9,130 | 1890 | 0.21 | 4.7 |
| 3 | 734 | 504 | 1,238 | 7,340 | 5,040 | 12,380 | 617 | 4,423 | 11,763 | 12,380 | 2689 | 0.23 | 4.4 |
| 4 | 1,035 | 606 | 1,641 | 10,350 | 6,060 | 16,410 | 639 | 5,421 | 15,771 | 16,410 | 2930 | 0.19 | 5.4 |
| 5 | 854 | 642 | 1,496 | 8,540 | 6,420 | 14,960 | 781 | 5,639 | 14,179 | 14,960 | 3184 | 0.22 | 4.5 |
| 6 | 413 | 816 | 1,229 | 4,130 | 8,160 | 12,290 | 1,224 | 6,936 | 11,066 | 12,290 | 2169 | 0.20 | 5.1 |
| 7 | 963 | 1,447 | 2,410 | 9,630 | 14,470 | 24,100 | 2,004 | 12,466 | 22,096 | 24,100 | 4665 | 0.21 | 4.7 |
| 8 | 244 | 640 | 884 | 2,440 | 6,400 | 8,840 | 1,104 | 5,296 | 7,736 | 8,840 | 1701 | 0.22 | 4.5 |
| 9 | 139 | 468 | 607 | 1,390 | 4,680 | 6,070 | 650 | 4,030 | 5,420 | 6,070 | 861 | 0.16 | 6.3 |
| 10 | 136 | 383 | 519 | 1,360 | 3,830 | 5,190 | 539 | 3,291 | 4,651 | 5,190 | 838 | 0.18 | 5.6 |
| 11 | 627 | 150 | 777 | 6,270 | 1,500 | 7,770 | 152 | 1,348 | 7,618 | 7,770 | 1715 | 0.23 | 4.4 |
| 12 | 615 | 708 | 1,323 | 6,150 | 7,080 | 13,230 | 893 | 6,187 | 12,337 | 13,230 | 2799 | 0.23 | 4.4 |
| 13 | 225 | 631 | 856 | 2,250 | 6,310 | 8,560 | 829 | 5,481 | 7,731 | 8,560 | 1157 | 0.15 | 6.7 |
| 14 | 273 | 962 | 1,235 | 2,730 | 9,620 | 12,350 | 1,669 | 7,951 | 10,681 | 12,350 | 1903 | 0.18 | 5.6 |
| 15 | 868 | 862 | 1,730 | 8,680 | 8,620 | 17,300 | 927 | 7,693 | 16,373 | 17,300 | 3287 | 0.20 | 5.0 |
| 16 | 506 | 225 | 731 | 5,060 | 2,250 | 7,310 | 260 | 1,990 | 7,050 | 7,310 | 1646 | 0.23 | 4.3 |
| 17 | 413 | 938 | 1,351 | 4,130 | 9,380 | 13,510 | 1,544 | 7,836 | 11,966 | 13,510 | 1830 | 0.15 | 6.5 |
| **Bplus Total** | **9,036** | **10,557** | **19,593** | **90,360** | **105,570** | **195,930** | **14,593** | **90,977** | **181,337** | **195,930** | **36,360** |  |  |
| **Pre Bplus Period** | | | | | | | | | | | | | |
| **Site** | **All records** | **Sampled positive records** | **Sampled non-positive records** |  |  |  | **Total positive records** |  | **Total non-positive records** | **Total records** | **Sampled non-positive records** | **Probability of being selected** | **Weight** |
| 1 | 5,522 | 593 | 528 |  |  |  | 593 | - | **4,929** | 5,522 | 528 | 0.11 | 9.3 |
| 2 | 5,603 | 156 | 153 |  |  |  | 156 |  | 5,447 | 5,603 | 153 | 0.03 | 35.6 |
| 3 | 9,439 | 536 | 506 |  |  |  | 536 |  | 8,903 | 9,439 | 506 | 0.06 | 17.6 |
| 4 | 6,182 | 343 | 321 |  |  |  | 343 |  | 5,839 | 6,182 | 321 | 0.05 | 18.2 |
| 5 | 8,225 | 462 | 435 |  |  |  | 462 |  | 7,763 | 8,225 | 435 | 0.06 | 17.8 |
| 6 | 13,046 | 1,400 | 900 |  |  |  | 1,400 |  | 11,646 | 13,046 | 900 | 0.08 | 12.9 |
| 7 | 7,466 | 637 | 580 |  |  |  | 637 |  | 6,829 | 7,466 | 580 | 0.08 | 11.8 |
| 8 | 8,017 | 1,040 | 906 |  |  |  | 1,040 |  | 6,977 | 8,017 | 906 | 0.13 | 7.7 |
| 9 | 4,657 | 657 | 563 |  |  |  | 657 |  | 4,000 | 4,657 | 563 | 0.14 | 7.1 |
| 10 | 4,454 | 602 | 520 |  |  |  | 602 |  | 3,852 | 4,454 | 520 | 0.13 | 7.4 |
| 11 | 4,544 | 83 | 80 |  |  |  | 83 |  | 4,461 | 4,544 | 80 | 0.02 | 55.8 |
| 12 | 6,686 | 509 | 468 |  |  |  | 509 |  | 6,177 | 6,686 | 468 | 0.08 | 13.2 |
| 13 | 2,731 | 365 | 317 |  |  |  | 365 |  | 2,366 | 2,731 | 317 | 0.13 | 7.5 |
| 14 | 16,980 | 2,674 | 2,245 |  |  |  | 2,674 |  | 14,306 | 16,980 | 2,245 | 0.16 | 6.4 |
| 15 | 6,337 | 520 | 477 |  |  |  | 520 |  | 5,817 | 6,337 | 477 | 0.08 | 12.2 |
| 16 | 6,545 | 296 | 254 |  |  |  | 296 |  | 6,249 | 6,545 | 254 | 0.04 | 24.6 |
| 17 | 8,776 | 1,184 | 1,025 |  |  |  | 1,184 |  | 7,592 | 8,776 | 1,025 | 0.14 | 7.4 |
| **Pre Bplus Total** | **125,210** | **12,057** | **10,278** |  |  |  | **12,057** |  | **113,153** | **125,210** | **10,278** |  |  |
| Totals preBplus+Bplus |  |  |  |  |  |  | **26,650** |  | **294,490** | **321,140** | **46,638** |  |  |
